# The Safety of Drug Treatment in Patients with Neuropathic Pain: Data from Ambulatory Care in a Real-Life Setting

**DOI:** 10.3390/reports6040057

**Published:** 2023-12-01

**Authors:** Cristina Vocca, Vincenzo Rania, Antonio Siniscalchi, Caterina Palleria, Gianmarco Marcianò, Cecilia Galati, Luca Catarisano, Valentina Mastrangelo, Franco Corasaniti, Francesco Monea, Lucia Muraca, Rita Citraro, Bruno D’Agostino, Luca Gallelli, Giovambattista De Sarro

**Affiliations:** 1 Pain Medicine Room, Complex Operative Unit of Clinical Pharmacology and Pharmacovigilance, Renato Dulbecco University Hospital, 88100 Catanzaro, Italy; cristina_vocca@live.it (C.V.); raniavincenzo1@gmail.com (V.R.); palleria@unicz.it (C.P.); gianmarco.marciano3@gmail.com (G.M.); lucacatarisano@gmail.com (L.C.); valentina.mastrangelo.vm@gmail.com (V.M.); citraro@unicz.it (R.C.); desarro@unicz.it (G.D.S.); 2 Department of Neurology and Stroke Unit, Annunziata Hospital of Cosenza, 87100 Cosenza, Italy; anto.siniscalchi@libero.it; 3 Research Center FAS@UMG, Department of Health Science, Magna Graecia University, 88100 Catanzaro, Italy; galaticecilia86@gmail.com (C.G.); francescomonea@libero.it (F.M.); 4 Department of Primary Care, ASP Catanzaro, 88100 Catanzaro, Italy; francocorasanitim@gmail.com (F.C.); lalumuraca@gmail.com (L.M.); 5 Department of Health Science, Magna Graecia University, 88100 Catanzaro, Italy; 6 Department of Environmental Biological and Pharmaceutical Sciences and Technologies, University of Campania “Luigi Vanvitelli”, 81100 Caserta, Italy; bruno.dagostino@vanvitelli.it; 7 Medifarmagen SRL, Renato Dulbecco University Hospital, 88100 Catanzaro, Italy

**Keywords:** neuropathic pain, adverse drug reactions, clinical records, pharmacovigilance

## Abstract

Introduction: Drug treatment can be related to the development of adverse drug reactions (ADRs). Aim: In this paper, we evaluated ADRs in patients admitted to the Ambulatory of Pain Medicine of the University Hospital Renato Dulbecco in Catanzaro. Methods: We conducted a prospective analysis between 1 February 2021 and 20 July 2023 on patients with neuropathic pain referred to the Ambulatory of Pain Medicine of “Renato Dulbecco” University Hospital in Catanzaro (Calabria, Italy). Patients aged >18 years with clinical signs of neurologic pain and a score upon completing the Douleur Neuropathique en 4 Questions (DN4) questionnaire of ≥4 were included. The association between drugs and ADR or between drugs and drug–drug-interactions (DDIs) was evaluated using Naranjo’s probability scale and Drug Interaction Probability Scale (DIPS), respectively. Results: During the study period, we analyzed 2370 patients referred to the ambulatory of pain medicine. After the evaluation of inclusion and exclusion criteria, 33.5% of patients were enrolled. All patients presented at least one comorbidity and daily used a mean of five drugs (range 3–11). Using the Naranjo score, the development of ADRs was documented in 112 patients (score 6). Using parametric and non-parametric statistical analysis, we failed to report an association between ADR and dosage or ADR and patient characteristics. Conclusion: Our results show the development of ADRs in 18% of patients with neuropathic pain. This low percentage of drug interaction could be a limitation in real life because it is probably due to the site of the study and the appropriate prescription of drugs. Therefore, it shows that it is necessary to motivate healthcare to pay attention to the prescription of drugs in poly-treated patients to reduce the risk of ADRs.

## 1. Introduction

The International Association for the Study of Pain (IASP) defines neuropathic pain as pain caused by a lesion or disease of the somatosensory nervous system [1].

In a recent review of international guidelines and recommendations for the pharmacological treatment of neuropathic pain [2], we reported that first-line drugs with a moderate-to-high quality of evidence and strong recommendation are tricyclic antidepressants (TCA, e.g., amitriptyline), antiepileptics (α2δ calcium channel unit blockers, pregabalin, and gabapentin), and serotonin noradrenaline reuptake inhibitors (SNRI: duloxetine and venlafaxine). Capsaicin 8% patches, lidocaine patches, and subcutaneous injections of botulinum toxin type A have weak recommendations and are indicated for peripheral neuropathic pain only [2]. Finally, even if opioids have not been recommended in the treatment of chronic non-cancer pain due to the development of serious adverse drug reactions (ADRs), some authors have suggested that tramadol could be used in the management of neuropathic pain [3,4,5,6,7].

ADRs represent a serious problem during the treatment of patients with pain (i.e., anticholinergic effects for tricyclic antidepressants [8], abuse and misuse of gabapentinoids in patients using opioids [9], and constipation during opioid treatment [10].

Meaadi et al. studied the safety and efficacy of gabapentinoids in the management of neuropathic pain as follows: a systematic review with the meta-analysis of randomized controlled trials.

These reduce patients’ compliance [11,12,13]. To reduce the development of ADRs, which could be also related to the dosage and the long duration of treatment, a non-pharmacological treatment has been suggested. Nutrients are commonly used in patients with pain, e.g., Acetyl-L-carnitine [14,15,16,17,18,19,20], palmitoylethanolamide (PEA) [21,22,23,24,25,26,27], and alpha-lipoic acid [28,29,30,31,32,33,34,35].

However, local techniques, e.g., high-intensity low-frequency-pulsed magnetic fields (diamagnetic therapy) or the administration of oxygen–ozone therapy, could represent an add-on treatment in the management of neuropathic pain [36,37,38,39,40].

The aim of this study was to evaluate, in a real-life ambulatory care study, both the development and the characteristics of ADRs in patients with neuropathic pain.

## 2. Materials and Methods

### 2.1. Study Design

We conducted a prospective study between 1 February 2021 and 20 July 2023 on patients with neuropathic pain who were referred to the Ambulatory of Pain Medicine of “Renato Dulbecco” University Hospital in Catanzaro (Calabria, Italy). The study, approved by the Ethics Committee (Calabria Centro: number 22/2021), was carried out according to the Good Clinical Practice guidelines and under the ethical principles of the Declaration of Helsinki. Before the beginning of this study, all participants signed a written informed consent form.

### 2.2. Inclusion and Exclusion Criteria

We enrolled patients aged >18 years with clinical signs of neurologic pain and a score from the Douleur Neuropathique en 4 Questions (DN4) questionnaire of ≥4. Patients with less than two clinical accesses to the ambulatory were excluded.

Exclusion criteria included current patients with nociceptive pain or nociplastic pain, patients with Alzheimer’s disease, and patients with active cancer. Moreover, patients who did not sign the informed consent were excluded.

### 2.3. Protocol

Consecutive patients were referred to the Ambulatory of Pain of Medicine of the University of Catanzaro for chronic pain, and were screened for neuropathic pain using both clinical tests and the DN4 questionnaire. In agreement with inclusion and exclusion criteria, patients were enrolled in this study and signed informed consent forms. During the admission, demographic data, comorbidity, polytherapy, the drug used and dosage, previous ADRs, and the intensity of pain (using the Numerical rating scale, NRS) were collected. Each patient was asked if he or she could evaluate his or her level of pain on a scale from 0 to 10, where 0 equaled an absence of pain and 10 indicated the maximum level.

The DN4 is a clinician-administered, neuropathic pain diagnostic questionnaire consisting of ten items grouped in four sections evaluating the quality of pain (burning, painful cold, electric shocks) and its association with abnormal sensations (tingling, pins and needles, numbness, itching). A score ≥ 4 was suggestive of neuropathic pain.

The ADRs correlated to the treatment were evaluated using the Naranjo probability scale, which is in agreement with our previous studies [41,42].

The Naranjo probability scale is a validated scale used to classify the probability that an adverse event is related to drug therapy based on a list of weighted questions, which examine factors such as the temporal association of drug administration and event occurrence, alternative causes for the event, drug levels, dose–response relationships and previous patient experience with the medication. A score of 1–4 suggested the possible correlation between the drug and ADR, a score of 5–8 represented a probable correlation, and a score >8 indicated a certain correlation [42,43,44].

Collected data were then stored in an Access database with security code protection.

### 2.4. Endpoints

The primary endpoint was the development of ADRs during the treatment of neuropathic pain. The secondary endpoint was the correlation between ADRs and age, comorbidity, and polytherapy in men and women with neuropathic pain.

### 2.5. Statistical Analyses

Data are presented as the mean ± standard deviation (SD). For categorical parameters, the chi-square test was used. Student’s t-test and the Kruskal–Wallis test were used for non-parametric variables. The Shapiro–Wilk test was used to evaluate the normality of distribution. Pearson’s test and Sperman’s test were used for the correlation study. Logistic regression was performed to evaluate the influence of different factors on pain levels. *p*-values < 0.05 were considered statistically significant. Statistical analysis was performed using SPSS 22.0 (International Business Machines Corporation, Armonk, NY, USA).

## 3. Results

### 3.1. Demographic and Clinical Characteristics

**Figure 1:**
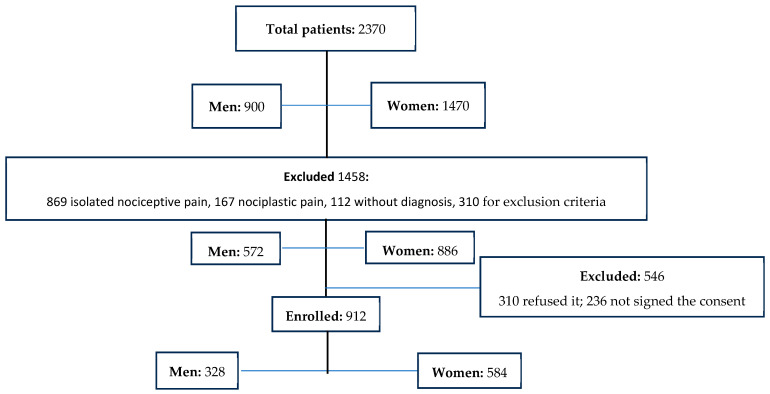
Flow chart showing enrolled patients.

During the study, we analyzed 2370 patients (men: 900, age 59.7 ± 11.6; women 1470, mean age 60.3 ± 11.9). After the evaluation of inclusion and exclusion criteria evaluation, 912 patients (38.5%, mean age 61.4 ± 13; 328 men and 584 women, mean age 60.6 ± 13.4 and 61.8 ± 12.9) with neuropathic pain were enrolled (Figure 1) (Table 1).

Statistical evaluation failed to show a significant difference between men and women with respect to age, instruction level, and smoking history (*p* > 0.05). Evaluating the patients stratified by age, we documented that 133 men (59.4%) and 251 women (57%) were enrolled in the group 18–64 years, while 195 men (40.6%) and 333 women (43%) were in the group > 65 years. Statistical evaluation did not document a significant difference between these groups for age, BMI, DN-4, NRS, degree, or smokers (Table 2).

Of the 912 enrolled patients, 886 patients (97.2%, mean age 61.7 ± 12.9) had at least one comorbidity (men 313, mean age 61.4 ± 13; women 573, mean age 61.9 ± 13); the most common were diabetes and osteoarthritis (Table 2). Psychiatric, rheumatologic, and orthopedic diseases were significantly more common in women (Table 2).

Concerning the comorbidity, we documented that diabetes was the most common comorbidity in the group 18–64 years (men 55.3%, women 55.4%, *p* > 0.05) and in elderly men (73.1%), while cardiovascular diseases and osteoarthritis were the most common comorbidities in elderly women (83.7%) (Table 3).

Moreover, we documented a statistically significant difference between men and women for the presence of urological diseases (elderly men’s group, *p* < 0.01), rheumatological diseases (women’s groups, *p* < 0.01), and psychiatric diseases (elderly women’s group, *p* < 0.01) (Table 2). All enrolled patients of both sexes and in both groups used drugs for pain treatment (Table 2 and Table 3).

### 3.2. ADRs

During the study, 164 patients (18%), including 67 men (40.7%) and 97 women (59.4%) developed ADRs (Table 4).

The statistical evaluation did not show any significant difference between men and women regarding age, BMI, DN4, degree, or if they were smokers (Table 4). Evaluating the comorbidities, we documented that women had a significant increase in neurological and rheumatologic diseases compared to men (*p* < 0.05) (Table 4).

Moreover, considering the gender differences between the patients with and without ADRs, we did not record any statistical difference between the patients who developed ADRs and those who did not (Table 5).

When we considered the difference between men and women with and without ADRs, we documented that smoking was most common in patients with ADRs compared to patients without ADRs (*p* < 0.01), without differences between men and women (Table 6). Considering the comorbidity, we recorded that rheumatological diseases and renal diseases were common in men with ADRs compared to men without (*p* < 0.01), while urological diseases were common in women with ADRs compared to women without (Table 7).

Considering the drugs involved in the development of ADRs, even if we documented a probable association between drugs and ADRs (Naranjo score: 6), we failed to record a significant correlation between the drug used and their dosages (Table 8).

We did not find any significant difference between the drugs used in patients with ADRs and patients without (Table 9).

The evaluation of the treatments involved in ADRs failed to identify a difference between men and women (*p* = 0.115). Moreover. the use of Pearson’s test failed to show a correlation between age, sex, degree, BMI, drug dosage, and ADRs (Table 10). The same result was recorded using multiple logistic regression analysis. The evaluation of ADRs documented that 1 patient (a man, smoker, 68-year-old, BMI 28.4) developed stypsis, confusion, and somnolence during polytherapy (Table 11). Similarly, six women developed more than one ADR during polytherapy.

Finally, the Pearson test did not show a correlation between polytherapy and ADRs (r: 0.02358).

## 4. Discussion

In this prospective study performed in the ambulatory care real-life setting, we evaluated both the types and the characteristics of ADRs to pharmacological and non-pharmacological treatments used to treat neuropathic pain.

Neuropathic pain is a chronic manifestation in which several clinical conditions (e.g., diabetes, inflammation, viral infections, injury) are able to induce a neuronal lesion with continuous neural activation.

Therefore, drugs able to modulate neural activation (e.g., channel blockers and modulators of neurotransmitter pathways) are commonly used [2].

In our study, we enrolled consecutive patients who were referred to the Pain Medicine Ambulatory of our University Hospital of General Practitioners for neuropathic pain. History and clinical evaluation documented that these patients (men and women) had suffered from low back pain or cervicobrachial pain for several years, and all these patients received treatment, commonly opioids since international guidelines suggest that opioids are not the first line of treatment. This could be related to the idea that drugs must be used for pain intensity management more than for the type of pain.

Moreover, a clinical evaluation documented that 92.3% of enrolled men and 100% of enrolled women presented a comorbidity, commonly in both sexes, diabetes, and osteoarthritis.

This agrees with data from the literature reporting that osteoarthritis and diabetes mellitus are associated in the same patients [45,46]. Louati et al. [47], in a systemic review, documented the high prevalence of osteoarthritis among patients with diabetes mellitus (29.5 ± 1.2%) and of diabetes mellitus among patients with osteoarthritis (14.4 ± 0.1%). Moreover, in our study, we recorded a high prevalence of urological diseases in men and rheumatological diseases in women, as also reported in data from the literature [48].

The presence of comorbidity or polytherapy must be considered in patients with pain because both diabetes and rheumatological diseases can impair pain levels. Similarly, the presence of urological or renal diseases can reduce renal activity; therefore, in these patients, the treatment must appropriate in order to reduce the risk of ADRs [49,50,51,52,53].

Both ADRs and inappropriate therapy represent a major concern in clinical practice because they can reduce adherence to the treatment [54,55,56,57,58,59,60,61], increasing health costs [49,62,63,64,65].

In this study, we documented that 18% of enrolled patients developed ADRs without a difference with respect to sex and without any correlation with respect to BMI, age, study, or other demographic characteristics. Moreover, we failed to report a correlation between ADRs and the DN4 score, and in all patients, the Naranjo score documented a probable association between drugs and ADRs.

The most common drugs involved in ADRs were opioids, and this is related to the low safety of these drugs. Moreover, it is important to remember that opioids are no longer recommended for the treatment of most patients with chronic pain. In fact, Nury et al. [66], reviewing data from the literature suggested that long-term opioid therapy (≥6 months) in chronic non-cancer pain may not be superior to nonopioids in improving pain or disability or pain-related functions but seems to be associated with more adverse events, opioid abuse or dependence, and possibly an increase in all-cause mortality.

We documented that opioid use induced the development of stypsis and mild CNS effects (e.g., somnolence and confusion), as described in drug labels [67,68,69,70]. Similar CNS effects were recorded during the treatment with pregabalin and antidepressants [71,72].

Pregabalin and duloxetine are commonly used in the management of neuropathic pain and represent the first line of treatment [2]. Their effects in the management of neuropathic pain can be related to the block of neural depolarization (pregabalin) and to the potentiation of the inhibitory pathway (duloxetine). These mechanisms induce the development of central side effects that could be reduced by starting with a low dosage and not using drugs that are able to induce CNS inhibition. In our study, we recorded that pregabalin was used with a high first dosage (125 or 150 mg daily) even if the total dosage was similar to the patients who did not develop ADRs. Duloxetine was co-administered with opioids that can induce CNS inhibition.

During this study, we reported fewer side effects in patients using nutraceuticals and oxygen–ozone therapy. These produce a very interesting result for physicians, especially considering the great efficacy of oxygen–ozone therapy [73,74,75,76,77,78].

Moreover, Magalhaes et al. [79], analyzing data from the literature in patients with low back pain secondary to disc herniation treated with oxygen–ozone therapy, documented both the safety and the efficacy of oxygen–ozone with evidence of the level II-3 for ozone therapy applied intradiscally and II-1 for ozone therapy applied paravertebrally (recommendation: 1C for ozone therapy applied intradiscally; 1B for ozone applied at the paravertebral muscles).

Diamagnetic therapy and acetyl-L-carnitine had no side effects, showing an excellent safety profile, according to previous experience and the label [36,80].

Our study has several limitations. Firstly, the number of patients is relatively small to draw definitive conclusions, and the number of women is high in comparison to men, even if the real-life setting is characterized by a high number of women coming to our ambulatory in comparison to men. Data were obtained in a clinical room of pain medicine where specialists in clinical pharmacology performed diagnosis and treatment, and this probably reduced the development of ADRs, also related to the comorbidity, polytherapy, level of instructions, and smoke. We used diamagnetic therapy, which is not a pharmacological treatment, but we reported it because we use it in some patients as an add-on therapy to reduce the dosage of drug treatment.

In conclusion, we documented that drugs used in the management of neuropathic pain are usually safe and, if prescribed appropriately, do not induce the development of severe ADRs or drug interactions.

## Figures and Tables

**Table 1:** Demographic characteristics at the time of enrollment. The data are expressed as a total number (N) and as a percentage (%) of enrolled patients (men: 328; women: 584).

	Men N: 328	%	Women N: 584	%	Delta Percentage
Age					
18–64	195	59.4	333	57.0	−4.2
≥65	133	40.6	251	43.0	6.2
Degree					
Yes	62	18.8	128	21.9	15.9
No	266	81.2	456	78.1	−3.7
Body mass index					
<25	82	25.0	184	31.6	26
25–30	169	51.6	200	34.2	−33.6
≥30	77	23.4	200	34.2	45.5
Smokers					
Yes (or former smokers)	190	57.8	194	33.3	−42.7
No	138	42.2	390	66.7	58.7
Diagnosis					
Low back pain	159	48.4	276	47.4	−2.5
Low back pain + cervicobrachial pain	149	45.3	272	46.5	2.6
Cervicobrachial pain	21	6.3	36	6.1	−3.1

**Table 2:** Characteristics of enrolled patients (men: 328; women: 584) stratified for age. Data referring to age, BMI, DN4, and NRS are expressed as the mean ± standard deviation. Other data are expressed as the total number (N) and percentage (%) of enrolled patients. BMI: body mass index; DN4: Douleur Neuropathique en 4 Questions. NRS: numerical rating scale. ** *p* < 0.01.

	18–64		>65		18–64		>65	
	Men				Women			
	N	%	N	%	N	%	N	%
Enrolled	195	59.4	133	40.6	333	57.0	251	43.0
Age	52.1 ± 9.8		73 ± 6		53.3 ± 9.2		73.1 ± 7	
BMI	27.4 ± 3.9		26.9 ± 4.7		27.5 ± 5.5		28.5 ± 5.2	
DN4	5.7 ± 1.1		5.8 ± 1.4		5.8 ± 1.2		5.9 ± 1.2	
NRS	8.2 ± 1.7		7.9 ± 1.3		8.3 ± 1.3		8.7 ± 1.4	
Degree	31	15.8	76	57.1	135	23.1	119	20.4
Smokers	103	52.8	133	100.0	216	64.8	151	60.2
Comorbidity	180	92.3	133	100.0	323	96.9	251	100.0
Cardiovascular diseases	82	42.1	87	65.4	164	49.2	213	84.8
Diabetes	108	55.4	97	72.9	184	55.4	184	73.3
Osteoarthritis	82	42.1	87	65.4	220	66.1	208	82.8
Urologic diseases	41	21.1	82	61.5 **	21	6.3	26	10.3
Gastrointestinal diseases	41	21.1	46	34.6	128	38.4	103	41
Neurological diseases	26	13.3	31	23.1	108	32.4	61	24.3
Rheumatological diseases	15	7.7	8	6	133	40.0 **	56	22.3 **
Psychiatric diseases	26	13.3	0	0.0	72	21.6	41	16.3 **
Renal diseases	21	10.8	21	15.7	10	3.1	36	14.3
Respiratory diseases	10	5.1	21	15.7	36	10.8	41	16.3
Hematological diseases	5	2.6	21	15.7	31	9.3	21	8.3
drug users	195	100.0	133	100.0	333	100.0	251	100.0

**Table 3:** Drug used in enrolled patients (men: 328; women: 584) stratified for age. Data are expressed as the total number (N) and percentage (%) of enrolled patients.

	18–64		>65		18–64		>65	
	Men				Women			
	N	%	N	%	N	%	N	%
**Opioids**								
Oxycodone/naloxone	30	9.1	11	3.4	15	2.6	26	4.5
Buprenorphine	12	3.7	3	0.9	35	6.0	26	4.5
Codeine	38	11.6	13	4.0	83	14.2	40	6.8
Tramadol	41	12.5	26	7.9	56	9.6	26	4.5
Tapentadol	4	1.2	4	1.2	6	1	9	1.5
Fentanyl	8	2.4	2	0.6	13	2.2	2	0.3
Oxycodone	10	3.0	15	4.6	11	1.9	9	1.5
**Antidepressants**								
Amitriptyline	12	3.7	13	4.0	30	5.1	16	2.7
Duloxetine	36	11	15	4.6	53	9.1	40	6.8
**Antiepileptics**								
Pregabalin	91	27.7	62	18.9	118	20.2	82	14
**Other treatments**								
Eperisone	35	10.7	27	8.2	52	8.9	51	8.7
Cannabidiol and β-caryophyllene	23	7.0	18	5.5	39	6.7	33	5.7
Cyclobenzaprine	14	4.3	11	3.4	35	6	11	1.9
Tizanidine	8	2.4	2	0.6	15	2.6	5	0.9
L-acetyl-carnitine	63	19.2	30	9.1	58	9.9	59	10.1
Nutraceuticals	97	29.6	77	23.5	179	30.7	154	26.4
Diamagnetic therapy	102	31.1	41	12.5	133	22.8	82	14
Oxygen–ozone therapy	151	46.0	85	25.9	220	37.7	153	26.2

**Table 4:** Demographic characteristics of enrolled patients who developed adverse drug reactions (ADRs) to drugs used in the management of neuropathic pain (men 67; women 97) The percentage of enrolled patients with ADRs was calculated with respect to the total number of enrolled patients (men: 328; women: 584). Data referring to age, BMI, DN4, and NRS are expressed as the mean ± standard deviation. Other data are expressed as the total number (N) and percentage (%) of enrolled patients. BMI: body mass index; NRS: numerical rating scale; DN4: Douleur Neuropathique en 4 Questions. * *p* < 0.05; ** *p* < 0.01.

	Men		Women		
	N	%	N	%	Delta Percentage
Enrolled	67	20.4	97	16.6	−18.7
Age	61 ± 13.2		60.3 ± 14		
BMI	27.3 ± 2.8		28.4 ± 5.4		
DN4	5.8 ± 1		6 ± 1.9		
NRS	9.3 ± 1.3		9.3 ± 1.1		
Degree	10	14.9	26	26.8	79.6
Smokers	41	61.2	46	47.4	−22.5
Comorbidity	62	92.5	97	100.0	8.1
Cardiovascular diseases	46	68.7	51	52.6	−23.4
Diabetes	41	61.2	56	57.7	−5.7
Osteoarthritis	31	46.3	71	73.2	58.2
Urologic diseases	21	31.3 *	10	10.3	−67.1
Gastrointestinal diseases	21	31.3	36	37.1	18.4
Neurological diseases	10	14.9	31	32.0 **	114.1
Rheumatological diseases	15	22.4	41	42.3 **	88.8
Psychiatric diseases	5	7.5	10	10.3	38.1
Renal diseases	15	22.4	10	10.3	−54.0
Respiratory diseases	5	7.5	15	15.5 **	107.2
Hematological diseases	10	14.9	15	15.5	3.6
drug users	67	100.0	97	100.0	0.0

**Table 5:** Student’s t-test evaluation in patients (men vs. women) with and without adverse drug reactions (ADRs). BMI: body mass index; DN4: Douleur Neuropathique en 4 Questions; NRS: numerical rating scale.

	Patients without ADRs	Patients with ADRs
	men vs. women	men vs. women
Age	0.233522	0.422397
BMI	0.208000	0.224596
DN4	0.430926	0.327175
NRS	0.061957	0.408738

**Table 6:** Gender differences in enrolled patients (men: 328 and women: 584) with or without adverse drug reactions (ADRs) during the treatment of neuropathic pain. Data referring to degree and smokers are expressed as the total number (N) and percentage (%) of enrolled patients; data referring to age, BMI, DN4, and NRS are expressed as the mean ± standard deviation of enrolled patients. BMI: body mass index; DN4: Douleur Neuropathique en 4 Questions. NRS: numerical rating scale. ** *p* < 0.01.

	Men				
	without ADRs		with ADRs		
	N	%	N	%	Delta Percentage
Enrolled	261	79.6	67	20.4	74.3
Age	60.5 ± 13.5		61 ± 13.2		
BMI	27.2 ± 4.6		27.3 ± 2.8		
DN4	5.8 ± 1.2		5.8 ± 1		
NRS	7.9 ± 1.5		9.3 ± 1.3		
Degree	13	5	10	14.9 **	199.6
Smokers	38	1.5	41	61.2 **	4024.1
	**Women**				
	without ADRs		with ADRs		
	N	%	N	%	Delta Percentage
Enrolled	487	83.4	97	16.6	80.1
Age	62.1 ± 12.6		60.3 ± 14		
BMI	27.9 ± 5.4		28.4 ± 5.4		
DN4	5.8 ± 1.1		6 ± 1.9		
NRS	8.3 ± 1.3		9.3 ± 1.1		
Degree	103	21.1	26	26.8	26.7
Smokers	149	30.6	46	47.4 **	55.0

**Table 7:** Enrolled patients (men: 328 and women: 584) with a comorbidity developing or not experiencing adverse drug reactions (ADRs) during the treatment of neuropathic pain. Data are expressed as the total number (N) or percentage (%) of enrolled patients. ** *p* < 0.01.

	Men				
	without ADRs (N)	%	with ADRs (N)	%	Delta percentage
Total	261	79.6	67	20.4	−74.3
Cardiovascular diseases	119	45.6	50	74.6	63.7
Diabetes	160	61.3	45	67.2	9.6
Osteoarthritis	135	51.7	34	50.7	−1.9
Urologic diseases	101	38.7	22	32.8	−15.1
Gastrointestinal diseases	65	24.9	22	32.8	31.8
Neurological diseases	46	17.6	11	16.4	−6.8
Rheumatological diseases	6	2.3	17	**25** .4 **	1003.7
Psychiatric diseases	20	7.7	6	9.0	16.9
Renal diseases	25	9.6	17	**25** .4 **	164.9
Respiratory diseases	25	9.6	6	9.0	−6.5
Hematological diseases	15	5.7	11	16.4	185.7
	**Women**				
	without ADRs (N)	%	with ADRs (N)	%	Delta percentage
Total	487	83.4	97	16.6	−80.1
Cardiovascular diseases	280	57.5	51	52.6	−8.6
Diabetes	317	65.1	56	57.7	−11.3
Osteoarthritis	372	76.4	71	73.2	−4.2
Urologic diseases	−24	−4.9	10	**10.** 3 **	−309.2
Gastrointestinal diseases	221	45.4	36	37.1	−18.2
Neurological diseases	133	27.3	31	32.0	17.0
Rheumatological diseases	158	32.4	41	42.3	30.3
Psychiatric diseases	72	14.8	10	10.3	−30.3
Renal diseases	36	7.4	10	10.3	39.5
Respiratory diseases	67	13.8	15	15.5	12.4
Hematological diseases	37	7.6	15	15.5	103.5

**Table 8:** Dosage used in enrolled patients with neuropathic pain that either developed or presented no drug reactions (ADRs). Data are expressed as mean ± standard deviation.

	Dosage		
Drug	Without ADRs	With ADR	*p* Value
Oxycodone	10 ± 7.1	45 ± 41.5	0.56357
Tramadol	78.8 ± 37.8	84.7 ± 54.4	0.86652
Buprenorphine	20.5 ± 26.0	26.9 ± 21.2	0.19847
Codeine	41.8 ± 17.0	30.0 ± 0	0.17533
Fentanyl	62.5 ± 17.7	41.7 ± 14.4	0.09469
Pregabalin	122.1 ± 65.3	138,49 ± 118	0.97648
Duloxetine	38.6 ± 13.9	38.6 ± 14.6	1
Amitriptyline	14 ± 12.8	24.5 ± 24.0	0.68413

**Table 9:** Treatments prescribed to enrolled patients (men: 328 and women: 584) with or without adverse drug reactions (ADRs) during the treatment of neuropathic pain. Data are expressed as the total number (N) and percentage (%) of enrolled patients.

	Men				
	without ADRs (n: 261)		with ADRs (n: 67)		
	N	%	N	%	Delta Percentage
**Opioids**	146				
Oxycodone/naloxone	26	10.0	15	22.4	124.7
Buprenorphine	10	3.8	5	7.5	94.8
Codeine	46	17.6	5	7.5	−57.7
Tramadol	46	17.6	21	31.3	77.8
Tapentadol	3	1.1	5	7.5	549.3
Fentanyl	5	1.9	5	7.5	289.6
Oxycodone	10	3.8	15	22.4	484.3
**Antidepressants**					
Amitriptyline	15	5.7	10	14.9	159.7
Duloxetine	41	15.7	10	14.9	−5.0
**Antiepileptics**					
Pregabalin	107	41.0	46	68.7	67.5
**Other treatments**					
Eperisone	26	10.0	36	53.7	439.4
Cannabidiol and β-caryophyllene	36	13.8	5	7.5	−45.9
Cyclobenzaprine	20	7.7	5	7.5	−2.6
Tizanidine	5	1.9	5	7.5	289.6
L-acetyl-carnitine	67	25.7	26	38.8	51.2
Nutraceuticals	143	54.8	31	46.3	−15.6
Diamagnetic therapy	97	37.2	46	68.7	84.7
Oxygen–ozone therapy	179	68.6	57	85.1	24.0
	**Women**				
	without ADRs (n: 487)		with ADRs (n: 97)		
	N	%	N	%	Delta Percentage
**Opioids**					
Oxycodone/naloxone	31	6.4	10	10.3	62.0
Buprenorphine	46	9.4	15	15.5	63.7
Codeine	97	19.9	26	26.8	34.6
Tramadol	56	11.5	26	26.8	133.1
Tapentadol	10	2.1	5	5.2	151.0
Fentanyl	5	1.0	10	10.3	904.1
Oxycodone	10	2.1	10	10.3	402.1
**Antidepressants**					
Amitriptyline	36	7.4	10	10.3	39.5
Duloxetine	67	13.8	26	26.8	94.8
**Antiepileptics**				0.0	
Pregabalin	154	31.6	46	47.4	50.0
**Other treatments**					
Eperisone	67	13.8	36	37.1	169.8
Cannabidiol and β-caryophyllene	36	7.4	36	37.1	402.1
Cyclobenzaprine	26	5.3	20	20.6	286.2
Tizanidine	10	2.1	10	10.3	402.1
L-acetyl-carnitine	97	19.9	20	20.6	3.5
Nutraceuticals	272	55.9	61	62.9	12.6
Diamagnetic therapy	154	31.6	61	62.9	98.9
Oxygen–ozone therapy	302	62.0	71	73.2	−15.7

**Table 10:** Pearson’s test correlation in patients with and without ADRs.

Patients without ADRs						
	BMI–NRS	BMI–DN4	NRS–DN4	Age–NRS	Age–DN4	Age–BMI
men	0.1774	0.0791	0.2597	−0.0845	−0.0737	−0.0049
women	0.1183	0.0884	0.0419	0.2375	0.0297	0.1603
**Patients with ADRs**						
	BMI–NRS	BMI–DN4	NRS–DN4	Age–NRS	Age–DN4	Age–BMI
Men	0.2063	0.0539	0.1275	−0.3820	0.0917	0.1463
Women	0.3221	0.0576	0.2001	0.1716	0.1118	0.3215

**Table 11:** Types of adverse drug reactions (ADRs) recorded in treated patients (men 67, women 97) for the management of neuropathic pain. * The same patient with more ADRs during polytherapy. The women’s group *a, *b, *c, *d, *e, *f, representing six patients (a–f) that developed more than one ADR. Data are expressed as the total number (N) and the percentage (%) of enrolled patients.

	Men (n: 67)			Women (n: 97)		
	N	%	Type	N	%	Type
Oxycodone	5	7.7	Stypsis (5)	5	5.3	somnolence (4); somnolence (1) *a
oxycodone/naloxone	10	15.4	stypsis (1) *; confusion (9)	5	5.3	stypsis (5) *f
Buprenorphine	5	7.7	blood hypertension (5)	10	10.5	stypsis (5); skin rash (4); skin rash (1) *a
Codeine	5	7.7	Stypsis (5)	5	5.3	stypsis (4); stypsis (1) *b
Tramadol	0	0.0		10	10.5	blood hypertension (9), (1) *c
Tapentadol	0	0.0		0	0.0	
Fentanyl	5	7.7	Stypsis (5)	0	0.0	
amitriptyline	10	15.4	confusion (6); somnolence (4)	5	5.3	Confusion (5)
Duloxetine	5	7.7	Confusion (5)	15	15.8	confusion (8); somnolence (7)
Pregabalin	10	15.4	confusion (1) *; somnolence (9)	31	31.6	Somnolence (28),(1) *d, (1) *e, (1) *f
Cyclobenzaprine	21	30.8	somnolence (20), (1) *	20	21.1	somnolence (17), (1) *b; (1) *d; skin rash (1) *e
Nutrients	0	0.0		15	15.8	blood hypertension (1) *c; bowel dysfunction (13), (1) *f
Oxygen–ozone therapy	0	0.0		10	10.5	pain in the site of administration (10)

## Data Availability

The data presented in this study are available on request from the corresponding author. The data are not publicly available due to privacy.
